# Modeling the Bone Marrow Niche in Multiple Myeloma: From 2D Cultures to 3D Systems

**DOI:** 10.3390/ijms26136229

**Published:** 2025-06-27

**Authors:** Adele Bottaro, Maria Elisa Nasso, Fabio Stagno, Manlio Fazio, Alessandro Allegra

**Affiliations:** Division of Hematology, Department of Human Pathology in Adulthood and Childhood “Gaetano Barresi”, University of Messina, via Consolare Valeria, 98125 Messina, Italy; adelebottarp15@gmail.com (A.B.); mariaelisanasso@gmail.com (M.E.N.); manliofazio@hotmail.it (M.F.); aallegra@unime.it (A.A.)

**Keywords:** 3D culture models, bone marrow microenvironment, multiple myeloma, drug resistance, molecular mechanisms, spheroids, organoids, tumor–stroma interactions

## Abstract

Multiple myeloma is a hematologic malignancy characterized by the clonal proliferation of plasma cells within the bone marrow. The tumor microenvironment plays a crucial role in multiple myeloma pathogenesis, progression, and drug resistance. Traditional two-dimensional cell culture models have been instrumental in multiple myeloma research. However, they fail to recapitulate the complex in vivo bone marrow microenvironment, leading to limited predictive value for clinical outcomes. Three-dimensional cell culture models emerged as more physiologically relevant systems, offering enhanced insights into multiple myeloma biology. Scaffold-based systems (e.g., hydrogels, collagen, and Matrigel), scaffold-free spheroids, and bioprinted models have been developed to simulate the bone marrow microenvironment, incorporating key components like mesenchymal stromal cells, osteoblasts, endothelial cells, and immune cells. These models enable the functional assessment of cell adhesion-mediated drug resistance, cytokine signaling networks, and hypoxia-induced adaptations, which are often lost in 2D cultures. Moreover, 3D platforms demonstrated improved predictive value in preclinical drug screening, facilitating the evaluation of novel agents and combination therapies in a setting that better mimics the in vivo tumor context. Hence, 3D cultures represent a pivotal step toward bridging the gap between basic myeloma research and translational applications, supporting the development of more effective and patient-specific therapies.

## 1. Introduction

### 1.1. General Considerations for Multiple Myeloma

According to the World Health Organization (WHO), in 2022, there were nearly 20 million new cases of cancer worldwide, and it is expected that this number will increase to 35 million in 2050. These data highlight the urgency in understanding cancer biology and its mechanisms of starting and progression in order to develop more effective biomarkers for early diagnostics and novel therapeutic systems [1].

Multiple myeloma (MM) is one of the most frequent types of blood cancer worldwide, second only to lymphoma [2,3]. Men are slightly more likely to get MM, and the usual age at diagnosis is 65 years. Approximately 56% of patients with MM survive for five years after diagnosis [4]. Despite the recent introduction of several medications and treatment regimens for MM [4], relapses occur in most patients [5], and MM is mostly incurable [6]. Multiple myeloma is a B-cell blood cancer characterized by the abnormal proliferation of clonal plasma cells (PCs). PCs cause osteolysis by releasing growth factors inside the bone matrix and facilitate disease progression and expansion in the bone marrow, resulting in enhanced osteoclastic activity [7]. Additionally, MM may be difficult to target because of heterogeneous chromosomal aberrations and mutations, which make it difficult to treat. It is widely acknowledged that multiple myeloma almost always develops following a previous stage known as monoclonal gammopathy of undetermined significance (MGUS), which progresses into active disease approximately 1% annually [8,9]. It remains unclear why some premalignant PCs stay in a dormant state for decades, while others undergo malignant transformation. Although the gradual accumulation of genetic mutations is presumed to drive disease progression, high-risk alterations such as del(17p13) and t(4;14) have also been identified in MGUS patients [10]. Hence, additional biological or microenvironmental factors are likely to be involved in initiating the malignant phase of the disease [11,12]. The interaction with the bone marrow matrix, composed of collagen, chondroitin sulphate, and hyaluronic acid [13], is the key to PC-mediated damage and drug resistance. Moreover, myeloma cells create an immunosuppressive environment to sustain tumor survival. MM is commonly detected in elderly people during a period marked by immunosenescence, an age-associated decline in immune function that heightens vulnerability to infections and malignancies. Additionally, the tumor microenvironment in MM, along with repeated exposure to chemotherapy, further impairs immune responses as the disease progresses [14]. The purpose of this review was to examine how the use of three-dimensional (3D) cultures changed the method used to investigate MM biology.

### 1.2. Bioprinting: An Innovation in the Field of Regenerative Medicine

The integration of smart materials into additive manufacturing revolutionized the field of regenerative medicine. Traditionally, 3D-printed scaffolds have been limited by their static nature, often falling short of replicating the dynamic and adaptive behavior of living tissues. Kantaros and Ganetsos highlighted that four-dimensional (4D) printing marks a significant shift toward creating structures capable of adapting and evolving in sync with biological systems [15]. Several classes of smart materials, like shape memory polymers, are engineered to adopt temporary configurations during the printing process and later revert to their original shapes when exposed to specific stimuli, such as body temperature. These materials show great promise in developing self-deploying implants tailored to individual anatomical and physiological needs. Liquid crystal elastomers are also discussed for their ability to mimic the anisotropic behavior of native tissues, responding dynamically to heat or light to guide cell alignment and tissue regeneration. Other materials, such as magnetic shape memory alloys, offer remote actuation through magnetic fields, enabling externally triggered shape changes or targeted drug delivery. Biodegradable polymers provide temporary structural support while gradually resorbing into the body, thereby avoiding secondary surgeries [15]. Particularly innovative is the use of cell-laden smart materials, which combine living cells with adaptive matrices to create constructs capable of biologically meaningful interactions with host tissue. Kantaros and Ganetsos [15] emphasized the immense potential of these materials for advancing regenerative therapies. From scaffolds that self-adjust to match the mechanical properties of growing tissue to minimally invasive devices that expand after implantation, the convergence of 4D printing and smart materials opens new avenues for personalized medicine. These developments might also enable sophisticated drug delivery platforms that respond to internal physiological cues, as well as fully biodegradable structures that eliminate the risks and complications of long-term implantation. However, before these technologies can achieve widespread clinical adoption, several challenges still remain. These include the need for enhanced printing precision, improved stability and reproducibility of smart materials, and rigorous biocompatibility testing.

A variety of 3D printing techniques are available, including fused deposition modeling (FDM), stereolithography (SLA), direct ink writing (DIW), and laser-guided direct writing (LGDW). These techniques provide benefits in terms of accuracy, material compatibility, and potential for use in biomedicine. They enable the fabrication of scaffolds and structures with precisely controlled shape, porosity, and mechanical characteristics, which are key factors for effective tissue regeneration. The selection of materials has an impact on biological outcomes. Thermoplastics like polycaprolactone (PCL) and poly(lactic acid) (PLA) provide mechanical strength and biodegradability. Sophisticated bioinks infused with cells or extracellular matrix components may also be used. Additionally, the material’s printability and its degradability must be carefully balanced to support cell viability and functionality [16].

Advancements in bioprinting technology hold significant promise for improving health-related quality of life by enabling the fabrication of patient-specific tissues and organs., Bioprinting facilitates the development of functional biological constructs tailored to individual needs through the precise control of cellular organization and material properties. It might also potentially reduce reliance on donor organs by minimizing immune rejection. All these innovations may lead to more effective regenerative therapies, faster recovery times, and improved outcomes in treating complex diseases. However, challenges still remain in ensuring the scalability, biocompatibility, and regulatory approval of bioprinted products before their clinical application [17].

### 1.3. Multiple Myeloma Biology: In Vivo Models

In vivo mouse models have been extensively utilized to gain deeper insights into the interactions between myeloma cells and their surrounding microenvironment. There are two types of mouse models: immunodeficient models are mainly used for drug discovery, and immunocompetent models are used to study the dissemination of neoplastic PCs and clinical signs such as bone lesions [18,19]. Immunosurveillance refers to the immune system’s ability to detect and eliminate foreign pathogens and cancer cells. Over the past fifteen years, this theory evolved into the notion of “immune editing”, and studies including a mouse model demonstrated the efficacy of immunosurveillance in controlling MM. In fact, natural killer cells and CD8^+^ T cells exert immune control through the interaction between CD226 (DNAM-1) and its ligand CD155 on malignant PCs [20]. Immunosenescence is one of the first causes of immunosurveillance loss. This phase leads, subsequently, to the onset of tumors. It is a multifactorial process that involves both a decrease in naïve T cells and an increase in proinflammatory cytokines such as TNF-α (tumor necrosis factor alpha), IL-1 (interleukin 1), and IL-6 (interleukin 6) [21]. Differently from what might be expected, the thymus in aged mice maintains the naïve T cell population, ensuring a sustained pool of these cells throughout their lifetime [22]. Beyond the decline in thymic output of naïve T cells, chronic antigen exposure also disrupts the balance between naïve and antigen-experienced T cell populations. This phenomenon has been observed in humans during ageing, persistent viral infections, and chronic malignancies. Notably, a significant shift toward a T cell population dominated by effector memory T (TEM) cells and CD8^+^ terminally differentiated effector memory RA (TEMRA) cells has been documented in human MM.

**Figure 1:**
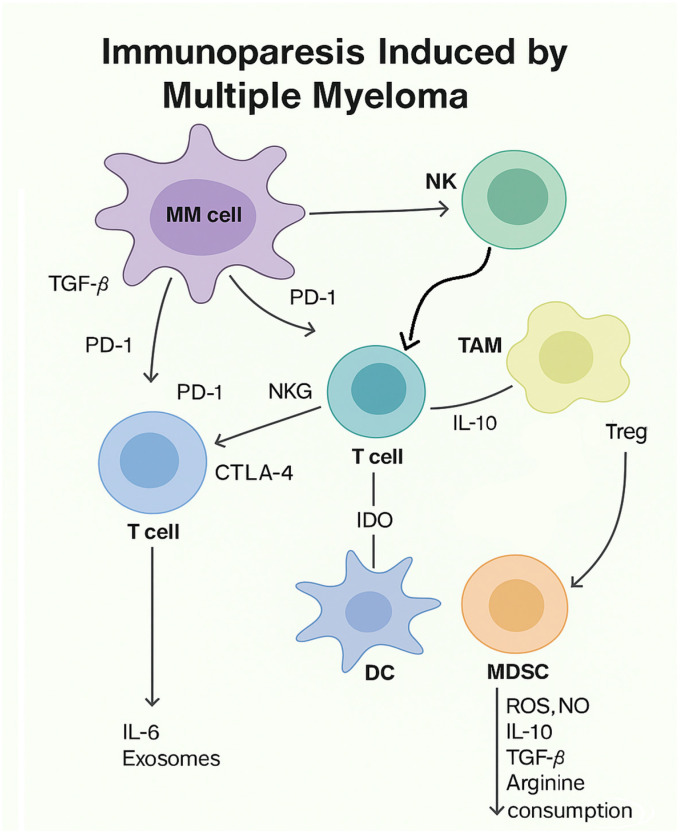
This figure illustrates how MM cells contribute to immunoparesis by secreting immunosuppressive factors such as TGF-β, IL-6, and PD-L1, which inhibit T and NK cell functions. Additionally, MM cells promote the expansion of regulatory T cells (Tregs), myeloid-derived suppressor cells (MDSCs), and tumor-associated macrophages (TAMs), further suppressing immune responses and facilitating disease progression.

MM is characterized by profound immune dysregulation, resulting in tumor-induced immunodeficiency and immunoparesis, as shown in Figure 1. Emerging evidence indicates that MM cells actively reshape the immune microenvironment to promote disease progression and immune evasion. Several studies have shown that myeloid-derived suppressor cells (MDSCs) and dysfunctional neutrophils are significantly expanded in MM patients compared to those with MGUS, contributing to T-cell anergy and suppression of anti-tumor immunity [23]. This myeloid impairment appears to be a key driver of the immunosuppressive landscape in MM. In addition to cellular immune suppression, MM is associated with immunoparesis, defined as the reduction in uninvolved immunoglobulin isotypes. Immunoparesis has been linked to increased infection risk and inferior progression-free survival (PFS), although its prognostic value for overall survival (OS) remains debated [24]. Mechanistically, immunoparesis is attributed to both clonal plasma cell expansion, which dominates the bone marrow niche, and to the disruption of normal B-cell development and function (Figure 1) [25]. The immunosuppressive environment in MM is further shaped by increased levels of regulatory T cells (Tregs), impaired cytotoxic T-cell responses, and dysregulated cytokine signaling [e.g., IL-6, transforming growth factor beta (TGF-β), and stromal cell-derived factor 1 (SDF-1)] [26]. These alterations collectively result in diminished immune surveillance and poor responsiveness to immunotherapeutic interventions. Several strategies have been explored to overcome this immune dysfunction, including checkpoint inhibitors, immunomodulatory drugs (IMiDs), anti-CD38 monoclonal antibodies, and Chimeric Antigen Receptor T-cell (CAR-T) cell therapies. However, tumor-induced immune suppression, especially through myeloid and B-cell dysregulation, continues to limit the full potential of these approaches [27].

Experiments involving both immunodeficient and immunocompetent mice have been essential for highlighting how novel immunotherapeutic agents exert their effects. A huge portion of preclinical research on immunotherapeutics has been conducted using immunodeficient mice. Xenograft models have been valuable in generating preclinical data regarding the development of immunotherapies for phase 1 human trials. Furthermore, revisiting these models provided insights into potential combination therapies and informed the design of phase 3 trials. However, the actual effects in humans are often less pronounced than those observed in preclinical studies. Medical oncology is shifting toward personalized care, and testing the efficacy of immunotherapeutic drugs in an ex vivo model before administration would be ideal. Humanized mice may offer a solution, although replicating normal T cell development with a human thymus is not feasible. Nevertheless, this limitation might be less relevant in MM due to thymic atrophy associated with immunosenescence. Mouse models remain essential for selecting drugs for clinical trials, as in vitro methods cannot, at this time, accurately predict clinical efficacy and toxicity. However, moving from studies using human MM cell lines in immunodeficient mice to evaluate immunotherapies is expected to yield more insightful preclinical results regarding the application of both immunotherapies and the small molecule inhibitors. Notably, recognizing the complementary roles of the innate and adaptive immune systems, as well as the crucial role of dendritic cells, will be vital for advancing the success of combination immunotherapies [28,29]. Innovative platforms, such as 3D bone marrow culture models, are being used to better understand immune–tumor interactions and improve drug screening.

## 2. Three-Dimensional Cultures: Innovation in the Field of Research

### General Considerations for 3D Cultures

Despite the wealth of data generated from molecular biology studies, a major challenge in cancer research remains the lack of experimental models that accurately replicate tumor biology and treatment responses. Historically, research relied heavily on two-dimensional (2D) cell culture systems, which fail to mimic the complexity of native human tumor tissues [30]. The tumor microenvironment (TME) in humans possesses unique physical and chemical characteristics that are not fully recapitulated by either in vivo animal models or traditional 2D cultures. To reduce the gap between patient-specific tumor responses and experimental observations, more advanced models—namely, spheroids and organoids—have been developed. These 3D systems more closely resemble in vivo conditions, offering improved modeling of drug response, nutrient and oxygen gradients, cell–cell and cell–matrix interactions, as well as the spatial architecture of tumors [30,31].

**Figure 2:**
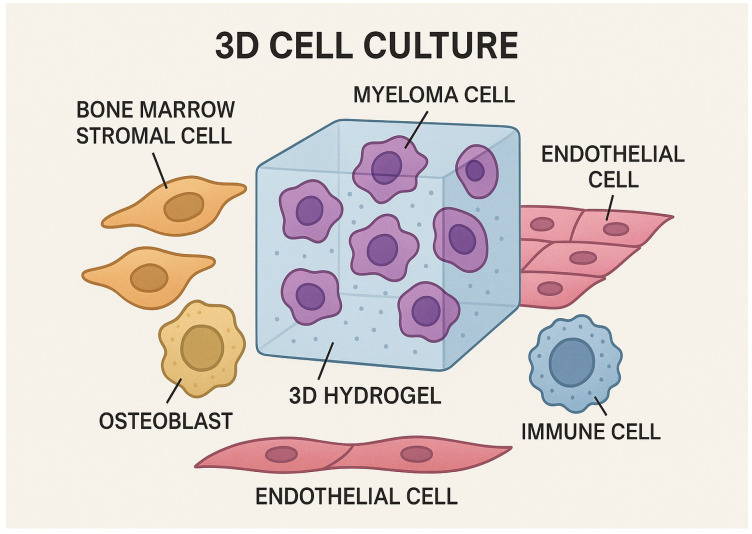
Schematic representation of a 3D cell culture model mimicking the bone marrow microenvironment in multiple myeloma. Myeloma cells are embedded within a 3D hydrogel scaffold that simulates the extracellular matrix.

It is widely accepted that 3D models (Figure 2) hold promise as substitutes for traditional preclinical models. Depending on the native cell material from which they are derived, these sophisticated 3D models are typically divided into spheroids or organoids. Spheroids are basic cell clusters that can be generated from various sources, such as primary cells, tissue samples, or established cell lines [32]. These cells group together to form spherical, compressed formations that serve no particular purpose. On the other hand, stem cell-derived organoids can form intricate 3D clusters that self-organize and differentiate into tissue-specific cell types, which frequently produce self-renewing structures that resemble organs and perform comparable activities. The most widely used 3D models in tumor research are spheroids because they are straightforward. These are tiny clusters of densely packed cells that can derive from a variety of tumors [2,33]. Spheroids can be homotypic, being made entirely of tumor cells, or heterotypic, being made up of a variety of cell types, such as fibroblasts, immune cells, or endothelial cells. In an in vitro three-dimensional setting, cells dissociate and re-aggregate to create clusters that can self-organize and develop into various cell types, a process known as organoid creation. These clusters closely resemble real organs and reflect characteristics of the in vivo microenvironment. Primary tissue biopsies, adult stem cells (ASCs, also known as primary tissue stem cells), embryonic stem cells (ESCs), and induced pluripotent stem cells (iPSCs) are some of the sources from which organoids can be produced [34]. Although the organoids may have different origins, they all go through a similar creation process. In general, this includes an initial tissue dissociation into single cells or clusters, after which they are embedded in a three-dimensional matrix (such as Matrigel) and cultivated in rich media enhanced with particular growth and differentiation factors (e.g., fibroblast growth factors, R-spondin, Noggin, retinoic acid, and epidermal growth factor) [35]. Three-dimensional cultures are increasingly gaining importance in the field of hematological diseases, not only in MM but also, for example, in chronic lymphocytic leukemia (CLL) [36]. A study by Belloni et al. described a detailed protocol for constructing 3D bone marrow (BM) surrogate microenvironments using a rotary cell culture system (RCCS™) to model the niche supporting CLL and MM. This dynamic 3D culture method involves the use of gelatin-based scaffolds pre-seeded with human bone marrow stromal cells (HS-5) and subsequently populated with tumor cells (MEC-1 for CLL, MM.1S for MM). The scaffolds were cultured in High Aspect Ratio Vessels (HARVs) within the RCCS™ bioreactor, which simulates microgravity and enhances nutrient and gas exchange, thereby supporting cell viability and mimicking the in vivo BM architecture. This system effectively recapitulates tumor–stroma interactions and allows for the evaluation of drug responses, such as the mobilization of CLL cells by ibrutinib and apoptosis induction in MM cells by bortezomib. The protocol includes precise instructions for scaffold preparation, static and dynamic cell seeding, culture maintenance, drug administration, and downstream analyses (e.g., flow cytometry, confocal microscopy, Western blotting, qPCR, and ELISA). Furthermore, the model can be extended to incorporate primary patient-derived cells and additional microenvironmental components, including endothelial and immune cells. Despite limitations related to vessel volume, cost, and scaffold variability, this 3D platform offers a versatile and physiologically relevant tool to investigate tumor biology and assess therapeutic efficacy in a controlled in vitro setting [36].

Most in vitro studies are performed in 2D in vitro cultures. Two-dimensional cell cultures remain a valid tool to investigate MM, particularly in the early phases of drug screening and therapeutic validation. These models, characterized by their reproducibility, scalability, and simplicity, continue to play a crucial role in preclinical research. One of the earliest investigations into optimal culture conditions for MM cells demonstrated that 2D monolayer systems, especially when supplemented with bone marrow stromal cells (BMSCs) and growth factors, can sustain the survival and proliferation of both myeloma cell lines and primary patient-derived cells for extended periods (up to 14 days), providing a robust platform for pharmacological testing [37]

More recent efforts have refined co-culture strategies, enhancing the physiological relevance of 2D models. Cornelis et al. introduced a straightforward and highly reproducible co-culture method combining primary MM cells with BMSCs in a 2D format, allowing for drug sensitivity testing with minimal cell numbers and no need for fluorescent labeling [38]. The importance of 2D cultures in early-phase validation is further supported by comparative studies evaluating multiple culture formats. While 3D-bioprinted constructs and transwell systems offer structural complexity, traditional 2D models remain the most practical and informative for initial drug sensitivity assays. This is relevant for standard-of-care agents like bortezomib and melphalan, where early IC_50_ values obtained in 2D systems can guide later-stage validations in more complex environments [39]. Furthermore, in ex vivo drug sensitivity profiling, Giliberto et al. used the short-term stimulation of patient-derived CD138^+^ MM cells in 2D cultures to evaluate synergistic drug combinations. Despite this ex vivo nature approach, the use of flat culture systems allowed for high-throughput screening while preserving the cells’ native phenotype and therapeutic response profiles [40]. However, this completely disregards the complexity of the interaction between the tumor cells and the microenvironment. Two-dimensional cultures have more surface contact with plastic than with other cells [41], so they are unable to recreate physiological conditions. Three-dimensional models promise to be more realistic in replicating cell-to-cell and cell-to-matrix interaction, even though they may alter cell proliferation, gene expression, and cell migration [42,43,44]. In vitro cultured cells may also change morphology according to the growing conditions. For example, 3D-grown cells may show enhanced elongation compared to 2D-grown cells [45] as well as a different morphology. Overall, cell aggregates or spheroids have been demonstrated to more accurately replicate native tissue architecture, particularly by mimicking the limited exposure of all cells to nutrients or pharmacological agents. The cellular composition of spheroids varies with their size. Proliferative cells typically occupy the peripheral layer, quiescent cells are found in the intermediate zone, and a necrotic core forms in larger spheroids (generally exceeding 500 μm in diameter) as a result of restricted nutrient availability, increased acidity, and waste accumulation. Consequently, the formation of such three-dimensional aggregates enhances the physiological relevance of in vitro models by establishing distinct cellular microenvironments. Even when spheroids deviate from a perfectly spherical morphology—potentially developing multiple necrotic regions—they still offer diverse microenvironments within the culture, which may influence cellular responses to external stimuli [46]. Drug intake may also differ between 2D and 3D cultures. Imamura et al. reported spheroids resistant to paclitaxel and doxorubicin when compared to looser spheroids or 2D growth. The denser spheroids showed decreased apoptosis and stained positive for Ki67 while having hypoxic centers. The results showed that a culture might be resistant to a certain drug depending on cell-to-cell and cell-to-matrix interaction in a 3D environment [45]. The alterations resulted from the difference in architecture between 2D and 3D cultures [46], as shown in Table 1. It is believed that cell morphology and interactions between cells may play a role in expression and treatment outcomes.

The table depicts the differences between 2D cell culture models and 3D cell culture models. Two-dimensional cultures show a low predictive value for clinical outcomes, as well as absent or highly reduced immune interaction modeling. Key pathways are also often not activated in 2D cultures. However, while 3D models appear to be more physiologically relevant than 2D models, costs are certainly higher and might increase with organ–on-chip automation. In conventional 2D cell culture systems, the absence of a three-dimensional (3D) microenvironment and structural support can significantly impact cellular behavior. For example, normal epithelial cells often lose their ability to differentiate and begin to exhibit cancer-like characteristics when cultured as a 2D monolayer. In contrast, cells grown in 3D environments display distinct differences in various aspects, such as morphology [47], proliferation [48], and functionality [49]. Beyond spatial configuration, cells in vivo are also exposed to dynamic conditions, constantly subjected to mechanical forces generated by blood flow, interstitial flow, and bodily movements. However, most current cell culture techniques—whether 2D or 3D—offer only static conditions, lacking mechanical stimulation. This is a critical limitation, as mechanical clues alongside biochemical signals are essential for regulating cell behavior. In the body, cells are exposed to a variety of mechanical forces, including tension, compression, and shear stress, all of which can profoundly influence cellular responses. For instance, myocardial cells experience rhythmic tension from heartbeats [50], blood cells are continuously subjected to shear stress as they circulate [51], and the transitional epithelium of the bladder endures pressure from urine accumulation Therefore, it is crucial to examine how cell behavior is different not only between 2D and 3D culture environments but also between static and dynamic conditions. Table 2 provides a short summary of the advantages and limitations of static and dynamic cultures.

## 3. Three-Dimensional Cultures in Multiple Myeloma

Many 3D models have been used to study the biology of MM. Two innovative 3D models, the liquid overlay and collagen-based systems, have been investigated as tools for studying MM in vitro [52]. The liquid overlay system allows MM cells to grow in a 3D configuration on a layer of liquid medium. This setup encourages the formation of cell clusters, or multicellular spheroids, which resemble tumor masses in vivo. The simplicity of the liquid overlay model makes it easy to use and reproducible, becoming an advantage for studies that require large-scale testing or high-throughput screening. In this model, the MM cells formed multicellular aggregates that exhibited characteristics of more aggressive tumor behavior, such as enhanced proliferation and resistance to cell death, as often seen in patients. These results suggest that the liquid overlay model is effective in mimicking the growth patterns and biological features of MM. The second model is the collagen-based 3D system, which is designed to more closely mimic the bone marrow microenvironment. Collagen, a fundamental component of the extracellular matrix (ECM), supports cell adhesion, migration, and differentiation. By embedding MM cells in a collagen matrix, researchers created a more physiologically relevant model that better reflects the conditions under which MM cells interact with the bone marrow stroma. This collagen-based environment allows MM cells to thrive, survive for extended periods, and exhibit drug resistance, as in patients. The model also illustrated how MM cells interact with the surrounding stromal cells, which is critical to disease progression. These interactions are known to promote MM cell survival, proliferation, and resistance to treatment, making the collagen-based model particularly useful for studying the tumor microenvironment and testing new therapies [52]. One of the most significant findings is the crucial role of tumor–stroma interactions in MM. Both the liquid overlay and collagen-based models showed how MM cells rely on the bone marrow stromal cells for survival and drug resistance. Stromal cells provide essential support through direct cell-to-cell contact and the secretion of soluble factors, such as cytokines, which enhance the survival and drug resistance of MM cells. This is particularly important because these tumor–stroma interactions often contribute to the failure of treatments in MM. This study underscores the need to develop therapies that target these interactions, either by disrupting the communication between MM cells and stromal cells or by altering the ECM. Moreover, both the liquid overlay and collagen-based models offer a more accurate representation of the MM tumor microenvironment, making them ideal platforms for evaluating the efficacy of new treatment strategies. These models could also be used to study the mechanisms of drug resistance, allowing the identification of new molecular targets and biomarkers for therapeutic intervention. Table 3 summarizes the various materials that can be used in 3D models.

Another experimental study tried to recreate fibrin gels from the peripheral blood plasma samples. This was intended as an alternative to BM plasma-derived gels in order to generate simple and available patient sample-independent 3D culture conditions for the U266 cell line [46]. Fibrin gels were created from plasma obtained from fresh or frozen peripheral venous blood in the presence of calcium chloride and tranexamic acid for the culture of U266 cells in 3D structures [53,54]. To evaluate the proliferation and vitality of cultivated cells, U266 cells without gels were used as control groups in the developed structures. U266 is one of the most used myeloma cell lines, which expresses IL-6, a crucial component for the maintenance and proliferation of myeloma cells. Flow cytometry was used to assess the expression of CD138 surface markers on U266 cells [46]. Jomehpour et al. [2] developed a cost-effective and physiologically relevant 3D culture system for the U266 multiple myeloma cell line using fibrin gels derived from peripheral blood plasma. In fact, researchers achieved optimal gel formation by using 1 mg/mL of calcium chloride and 5 mg/mL of tranexamic acid, thus producing stable and consistent fibrin matrices. Notably, the use of frozen plasma samples did not adversely affect gel formation or stability, enhancing the practicality of this method for widespread application. Within these fibrin-based 3D cultures, U266 cells demonstrated effective distribution and proliferation, indicating that the system successfully mimics aspects of the bone marrow microenvironment critical for MM pathophysiology. This model offers a more accurate in vitro platform for studying MM biology and evaluating therapeutic interventions, addressing limitations associated with traditional two-dimensional culture systems. Martini et al. [55,56] studied the long-term survival of plasma cells in a human bone marrow microphysiological 3D model created with a hydrogel-based system to simulate the physiological environment of the bone marrow niche. The aim of the study was to develop a more physiologically relevant model to maintain plasma cells for extended periods, offering insights into their behavior in the context of MM and other related conditions. The model is based on a hydrogel-enclosed 3D culture system, which encapsulates human bone marrow cells, including stromal cells, extracellular matrix components, and PCs. This system was developed to more closely simulate the bone marrow niche in vivo, providing a stable, dynamic microenvironment that supports the long-term viability and functionality of PCs. The research team was able to maintain viable and functional plasma cells for over 4 weeks in vitro, with cells continuing to produce immunoglobulins, which is a hallmark of PC activity. This innovative approach provides a more physiologically relevant platform for studying PCs’ behavior in the bone marrow niche, offering significant advancements in disease modeling and therapeutic development. The model has the potential to improve our understanding of PC biology and could be pivotal in advancing treatments for plasma cell-related diseases.

### Modeling Multiple Myeloma in 3D: Insights into Molecular Mechanisms

The BM microenvironment, in which MM PCs reside, is crucial to maintain their viability [57]. One of the most important pathways promoting the development of the disease is Wingless/integrated (WNT). The Wnt/β-catenin signaling pathway is a well-conserved molecular cascade that plays a key role in regulating vital biological functions, such as embryonic development, cell proliferation, tissue homeostasis, and the maintenance of stem cells. It has two primary branches: canonical (β-catenin-dependent) and non-canonical (β-catenin-independent) pathways. In the absence of Wnt ligands, cytoplasmic β-catenin is continuously targeted for degradation by a destruction complex comprising Axin, antigen-presenting cell (APC), Glycogen Synthase Kinase-3 beta (GSK-3β), and casein kinase 1 (CK1). Upon Wnt ligand binding to Frizzled (FZD) receptors and Lung Resistance-related Protein (LRP5/6) co-receptors, the destruction complex is inactivated, allowing β-catenin to accumulate and translocate to the nucleus, where it interacts with TCF/LEF transcription factors to regulate gene expression related to cell cycle progression and survival [57,58,59]. Non-canonical Wnt pathways, such as the planar cell polarity (PCP) pathway and the Wnt/Ca^2+^ pathway, function independently of β-catenin and are involved in regulating cytoskeletal organization, cell polarity, and morphogenetic movements. These branches are especially important during tissue patterning and organogenesis [59]. Dysregulation of Wnt signaling is implicated in the pathogenesis of various diseases. Hyperactivation of the canonical pathway, often due to mutations in APC or β-catenin (CTNNB1), is a hallmark of many cancers, notably colorectal carcinoma. On the other hand, insufficient Wnt signaling is associated with neurodegenerative diseases and impaired tissue regeneration [60]. Given its dual role in promoting proliferation and maintaining tissue integrity, the Wnt/β-catenin pathway is an attractive target for therapeutic modulation. Inhibitors of Wnt signaling are under investigation for cancer therapy, while Wnt agonists are being explored to enhance regenerative capacity in degenerative disorders [61]. Receptor Tyrosine Kinase-like Orphan Receptor 2 (ROR2), the receptor for the WNT non-canonical pathway, was found to be significantly expressed in myeloma cells by an objective evaluation of receptor tyrosine kinases overexpressed in myeloma. Moreover, it was found that its expression progressively increases going from MGUS to overt MM. The most prevalent growth factor in myeloma patients’ bone marrow is its ligand, WNT5A. In an in vivo model, reducing ROR2 (which supports the interaction between myeloma cells and the bone marrow microenvironment) caused the cells to detach from their niche and triggered apoptosis. An experiment was conducted to determine whether ROR2 has an effect on MM cells’ ability to home to the BM. It has been shown that the percentage of CD138^+^ cells in BM decreased significantly when ROR2 levels decreased. A simultaneous examination of the peripheral blood’s CD138^+^ cells showed that ROR2 knockdown significantly increased the number of circulating MM tumor cells. ROR2 activation leads to downstream signaling via the Phosphoinositide 3-kinase (PI3K)/protein kinase B (AKT)/mammalian target of rapamycin (mTOR) axis, promoting survival signals and anchorage of MM cells within the BM. Knockdown of ROR2 in MM cells results in impaired adhesion, increased apoptosis, and delayed disease progression in murine models, indicating its essential role in maintaining MM cell viability within their protective microenvironment. Importantly, pharmacological inhibition of PI3K/AKT signaling disrupted ROR2-mediated cell adhesion, suggesting a therapeutic vulnerability that can be exploited in ROR2-high MM patients. This study highlights ROR2 as a potential therapeutic target, particularly in high-risk MM subtypes where microenvironmental interactions are key drivers of disease persistence and relapse [62]. In contrast, the systematic review by Boulogeorgou et al. [63] characterizes the immune microenvironment of extramedullary MM lesions, where plasma cells disseminate and proliferate outside the protective BM niche. These extramedullary (EMD) sites display marked immune suppression, with an enrichment of exhausted CD8^+^ T cells, M2 macrophages, inactivated dendritic cells, and impaired NK cells, creating a permissive environment for tumor growth in the absence of classical BM support. Importantly, this extramedullary adaptation involves immune escape mechanisms that differ from the adhesion-mediated survival seen in BM-resident disease. Together, these studies illustrate a continuum of microenvironmental dependence in MM: while BM-localized myeloma relies on ROR2-mediated adhesion and PI3K/AKT signaling to interact with stromal and mesenchymal elements, EMD appears to exploit a distinct strategy, namely, the remodeling of the immune niche to suppress antitumor responses. This divergence underscores the plasticity of MM cells in adapting to spatially distinct microenvironments and suggests that therapeutic strategies must account for both stromal signaling and immune evasion mechanisms [63]. Both studies support a clinical rationale for combining ROR2/AKT-targeted agents with immune-modulatory therapies or checkpoint inhibitors [62,63]. Moreover, evidence suggests that increased cell survival, proliferation, and treatment resistance in MM are caused by proteins that Signal Transducer and Activator of Transcription 3 (STAT3) upregulates [64]. It is a transcription factor that plays a role in nearly all key characteristics of cancer, such as tumor growth, metastasis, angiogenesis, immune suppression, tumor inflammation, metabolic reprogramming, drug resistance, and cancer stemness [65]. Because this pathway is commonly active in primary MM cells, studies of MM in 3D culture methods can provide findings representative of the disease. The state of five pathways—STAT3; Extracellular Signal-Regulated Kinase (ERK)/mitogen-activated protein kinase (MAPK); PI3K/Akt; Nuclear Factor kappa-light-chain-enhancer of activated B cells (NF-κB); and Notch- in U266 and RPMI8226 cells, cultured in 3D or conventionally—has been investigated to evaluate whether the 3D culture shows a substantial effect on cellular signaling in MM cells [64]. It has been reported that MM-3D cells produced the active/phosphorylated form of STAT3 (pSTAT3), but cells cultivated conventionally did not. The kinetics of STAT3 activation in MM-3D cells were then investigated using a time course experiment. Western blot analysis was used every day to assess the expression level of pSTAT3 in cells from both MM cell lines grown in 3D for four days. The pSTAT3 band appeared in U266 cells on day 1 and on day 4, with a time-dependent increase in pSTAT3 that peaked. The pSTAT3 band was likewise visible in RPMI8226 cells on day 1, although it seemed to gradually fade thereafter. In contrast, cells cultivated using a conventional method did not exhibit any pSTAT3 bands. The expression levels of numerous cytokines that are known to cause STAT3 phosphorylation in MM, including IL6, IL21, and IL10, were studied to investigate potential STAT3 activators in 3D culture. After one day of 3D culture, U266 cells’ expression of all three cytokines rose 1.5–2.5 times more than that of cells in conventional culture [64]. In the same experimental study, it was also investigated whether STAT3 activity in MM-3D cells leads to resistance to bortezomib. It was demonstrated that Epidermal Growth Factor Receptor-induced (EGFR-induced) STAT3 activation increases resistance to proteasome inhibitors in MM cells [59]. Table 4 summarizes the characteristics of 3D cultures.

## 4. Multiple Myeloma Therapy: Applications of 3D Cultures

MM remains an incurable cancer, despite the development of several novel therapies. Significant therapeutic progress was made in recent years, particularly in relapsed or refractory cases. The development of 3D culture systems has been pivotal in advancing immunotherapy research, particularly in the context of CAR T-cell therapy. CAR T-cell therapies have shown promising results in treating MM, but their effectiveness is often limited by the complex tumor microenvironment, which can suppress immune responses. By using 3D models that replicate the stromal support and immune suppression seen in MM patients, researchers can more accurately assess the ability of CAR T-cells to infiltrate the tumor, interact with MM cells, and mediate cytotoxicity. These models allow for the optimization of CAR T-cell constructs and treatment protocols by providing a more predictive platform for evaluating immune cell-mediated killing in a physiologically relevant setting [65]. Other reports tried to understand how Suberoylanilide Hydroxamic Acid (SAHA), a histone deacetylase inhibitor (HDAC), might enhance the effect of Tumor Necrosis Factor-Related Apoptosis-Inducing Ligand (TRAIL) [66] in MM cells. TRAIL plays a critical role in regulating various cellular processes, e.g., immune surveillance, cell proliferation, and differentiation [67,68,69] by selectively inducing apoptosis [70] in tumor cells while sparing normal cells. This highlights its potential utility as a diagnostic, prognostic, and therapeutic response biomarker in oncology [71,72,73]. Moreover, TRAIL has been increasingly recognized as a promising therapeutic target. Research looked at how this combination could work in both traditional suspension cultures and more complex 3D cell culture models [74]. The study evaluated the combination of SAHA (at a concentration of 0.5 µM) and TRAIL (at 100 ng/mL) on six established human MM cell lines and one primary plasma cell leukemia culture. TRAIL successfully induced apoptosis in all the MM cell lines in a dose-dependent manner, reflecting that higher doses led to more cell death. The apoptosis-inducing effect of TRAIL was significantly enhanced when combined with SAHA in almost all the cell lines tested, except for the OPM-2 line. The combination treatment also activated key enzymes involved in the apoptotic process, such as caspase-8 and caspase-9. Interestingly, growth inhibition was observed when MM cells were treated with SAHA alone, and this effect was even more pronounced when SAHA was combined with TRAIL. In both suspension cultures and 3D cultures, the combination of SAHA and TRAIL reduced the number of viable cells. The 3D models, which were designed to simulate the in vivo conditions of tumors, showed that the combination of SAHA and TRAIL required lower doses to enhance the response compared to the 2D suspension cultures. However, the TRAIL-resistant cells, which were selected through exposure to TRAIL, did not respond further to the SAHA combination treatment. This suggests that while SAHA can sensitize many MM cells to TRAIL, it does not work on cells that have specifically developed resistance to TRAIL [75].

Another therapeutic option involves the possibility of targeting the microenvironment and, in particular, mesenchymal stromal cells (MSCs). MM is an aggressive hematologic cancer that suppresses osteoblast formation, mediated by bone marrow mesenchymal stromal cells (BM-MSCs), which can also support the growth and survival of PCs. The use of MSCs in MM therapy is a controversial topic due to conflicting results on the ability of MSCs to either inhibit or promote cancer growth. Previous studies have shown that MSCs can be loaded with paclitaxel (PTX) and used to deliver the drug in situ in sufficient amounts to affect tumor growth (both in vitro and in vivo) [75]. Hence, regardless of the debated action of MSCs in myeloma, MSCs could serve as a “Trojan horse” for delivering anti-cancer agents to the bone marrow.

In one study, BM-MSCs were loaded with paclitaxel and co-cultured with RPMI 8226 myeloma cells within a 3D dynamic culture system. The proliferation of the myeloma cells was then monitored to evaluate the treatment’s efficacy. As a result, a significant inhibition of myeloma cell growth was observed: PTXr-MSCs showed a dramatic suppression of myeloma cell growth, suggesting that drug-loaded MSCs could be a tool for delivering drugs to the bone marrow [76].

### 4.1. Three-Dimensional Models and Drug Resistance in Multiple Myeloma

**Figure 3:**
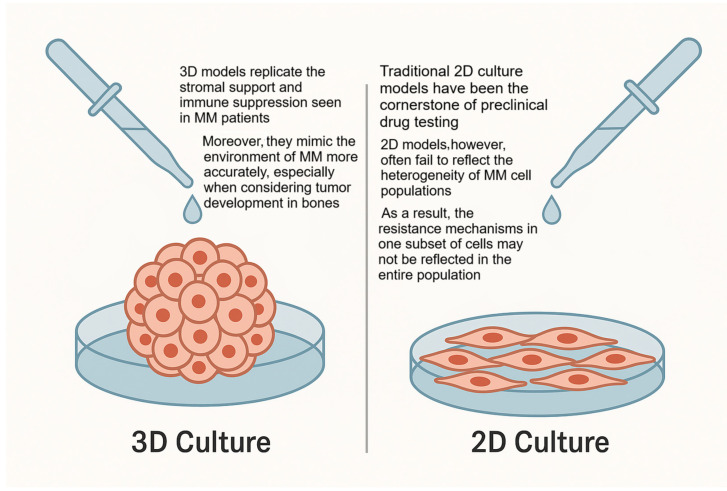
Three-dimensional models replicate the stromal support and immune suppression observed in MM patients. Moreover, they mimic the tumor microenvironment more accurately. While traditional 2D cultures have been central to preclinical drug testing, they often fail to capture the heterogeneity of MM cell populations.

To understand the complex and multifactorial nature of drug resistance in MM remains a major challenge. Traditional 2D culture models have been the cornerstone of preclinical drug testing, although they exhibit significant limitations in accurately mimicking the MM in vivo environment. Two-dimensional models often fail to reflect the heterogeneity of MM cell populations. In MM, clonal evolution results in a wide range of phenotypic and genotypic differences among tumor cells, which can contribute to differential drug responses [77]. In 2D cultures, however, the uniformity of cell growth does not capture the diversity of the tumor microenvironment, as summarized in Figure 3, or the interaction between the various subpopulations of MM cells. As a result, the resistance mechanisms in one subset of cells may not be reflected in the entire population. Another critical shortcoming of 2D cultures is their inability to accurately reproduce the cell adhesion-mediated drug resistance (CAM-DR) phenomenon. In vivo, MM cells are often tightly bound to the bone marrow stromal cells via adhesion molecules such as integrins and CD44, which provide survival signals that protect MM cells from cytotoxic drugs. These interactions are largely absent in 2D models where cells are detached from their natural ECM and stromal support [78]. Moreover, 2D cultures lack the oxygen and nutrient gradients that exist in tumors, and this can influence drug metabolism, cellular stress responses, and overall treatment efficacy [79]. Drug resistance is an unmet need when treating a patient with MM. Even with bortezomib treatment, which is one of the most effective drugs [80,81] against MM, PCs can still grow, showing resistance. Furthermore, bortezomib resistance has been observed even in naive patients who received the treatment for the first time [82]. There are many reasons for drug resistance: the inhibition of apoptosis, increased drug discharge or decreased uptake, and alterations in cycle-regulating factors. Another possible mechanism is the over-expression of the ATP-binding cassette, a transporter protein [83]. To prevent drug-induced apoptosis, the PCs activate an escape path by interacting with the stromal cells and the bone matrix, releasing growth factors as well as cytokines such as IL-6, IL-21, IGF-1, vascular endothelial growth factor (VEGF), TNF-alpha, SDF-, Rapidly Accelerated Fibrosarcoma (RAF)/mitogen-activated protein kinase (MEK)/MAPK [79]. Bortezomib acts against this mechanism by inhibiting the NF-kB pathway. NF-kB may also interfere with drug-induced apoptosis [84]. Unfortunately, while bortezomib blocks the canonical NF-kB pathway, it induces the non-canonical pathway, and myeloma cells become resistant to bortezomib anyway [85,86]. The microenvironment may cause drug resistance through cell adhesion and soluble factors, since IL-6 is linked to bortezomib resistance [87]. To overcome drug resistance, smart drug delivery methods have been studied; for example, nanoparticle-based delivery systems enable targeted and controlled release of chemotherapeutics, reducing side effects and enhancing therapeutic effects [88,89]. Nanoparticles used in therapeutic applications must be both biodegradable and biocompatible. Among various options, liposomes have shown significant promise due to their excellent biocompatibility. Targeted nanoparticles demonstrated particular potential as peptide-conjugated liposomes targeting CD38 and CD138 receptors, while chitosan-based nanoparticles loaded with bortezomib have been shown to enhance proteasome inhibition while minimizing side effects [90]. Nanoparticles can be classified by their composition, including polymer-based systems such as micelles and nanogels, or non-polymer-based systems like metallic nanoparticles. The physical and chemical characteristics of nanoparticles—such as size, shape, and surface properties—are largely determined by the materials used in their synthesis. Therefore, the rational design of nanoparticles is essential for achieving optimal therapeutic outcomes. A compelling example is the use of mesoporous silica nanoparticles loaded with bortezomib and functionalized to target the folic acid receptor on MM cells, which demonstrated significant efficacy in inducing apoptosis [91].

### 4.2. Controversies and Limitations in the Application of 3D Models

The use of 3D models has increased in the medical field. While these models offer significant advantages [92] in terms of visualization, simulation, and communication, their application is not without controversy or limitations. Some controversies might include ethical concerns. In fields such as medicine and forensics, the use of 3D models can raise ethical questions, especially when dealing with sensitive human data. Issues related to patient privacy, informed consent, and the potential misuse of biometric information are significant concerns [93]. Moreover, the creation and distribution of 3D models often involve questions about data ownership, copyright, and intellectual property. There is also an ongoing debate about the authenticity of 3D reconstructions. Critics argue that digital models may introduce interpretation biases, leading to inaccurate reconstructions. It is also quite difficult to replicate the same experiment twice while using 3D models [94]. Excessive reliance on 3D modeling can also lead to the undervaluation of traditional methods and skills. Moreover, technical malfunctions or obsolescence of software and file formats may compromise long-term accessibility. Despite technological advances, the creation of high-quality and accurate 3D models often requires sophisticated equipment, specialized knowledge, and substantial computational resources. These requirements can limit accessibility, especially in low-resource settings. In terms of accuracy, a 3D model depends heavily on the quality of the input data. Low-resolution scans or incomplete data can result in models that are misleading or insufficient for precise analysis. Last, but not least, producing detailed 3D models can be time-consuming and expensive [92,93]. This can be a limiting factor for time-sensitive or budget-constrained projects.

## 5. A Concise Overview on Organoids and Their Potential Role in Personalized Medicine: A Future Perspective

Organoids are simplified, miniature versions of internal organs cultivated in vitro, which have garnered significant interest in areas such as tissue development, disease modeling, clinical diagnostics, drug screening, and personalized medicine [95]. The successful development of physiologically relevant organoid cultures has notably propelled translational research in cancer therapy, an area previously hindered by discrepancies between two-dimensional cell cultures and in vivo studies [96]. In recent years, there has been an improvement in the success rate of organoid establishment, alongside an expansion in the variety of organoid types. Furthermore, the integration of advanced technologies, such as gene editing tools like Clustered Regularly Interspaced Short Palindromic Repeats and CRISPR-associated protein 9 (CRISPR-Cas9), has further extended the potential applications of organoids [97]. The concept of organoids originates from the discovery of the intrinsic self-organizing capacity of vertebrate cells. Remarkably, even after complete dissociation, these cells can re-aggregate and reconstruct the original architecture of the organ. As early as 1907, it was demonstrated that dissociated sponge cells possess the ability to reorganize into fully functional individuals [98]. This inherent cellular property led scientists to explore the potential of reconstructing partial or entire organs using tissue-derived or embryonic stem cells. Organoids represent a three-dimensional in vitro culture system that leverages this self-organizing behavior and can recapitulate the structural, functional, and genetic features of the native tissue [99]. In addition, organoids can be used to better understand tumors, including simulating their physiological and pathological behavior [100]. Their main property is to replicate many aspects of their in vivo counterparts. Usually, organoid cells are primarily derived from either pluripotent stem cells or tissue-specific progenitor cells. These stem cells are embedded within a suitable extracellular matrix and cultured in a defined medium containing a combination of growth factors that facilitate cellular self-organization and development. Under specific culture conditions, the stem cells are capable of self-organizing and proliferating into organ-like structures that can be continuously maintained over time while retaining a high degree of genetic stability. The standard components of the culture medium typically include advanced Dulbecco’s Modified Eagle Medium (DMEM)/F12, A83–01, primocin, penicillin/streptomycin, B27, N2, epidermal growth factor (EGF), Fibroblast Growth Factor 10 (FGF10), FGF7, 2-[4-(2-hydroxyethyl)piperazin-1-yl]ethanesulfonic acid (HEPES), Wnt3A, Noggin, GlutaMAX, hepatocyte growth factor (HGF), R-spondin-1, gastrin, prostaglandin E2, nicotinamide, neuregulin 1, N-acetylcysteine, Y27632 (a Rho kinase inhibitor), and SB202190 (a p38 MAPK inhibitor) [101]. There is a wide range of organoids, ranging from lungs to breast [75]. However, despite the significant potential of this technology in cancer research, several critical challenges continue to limit its application. Firstly, although three-dimensional organoids generated in vitro exhibit partial structural resemblance to native organs, their architecture remains relatively simplistic and can only approximate certain tissue characteristics [75]. Secondly, limitations inherent in current culture systems hinder the ability of organoids to fully replicate the physiological functions of whole organs. For instance, the use of ECM substitutes and fetal bovine serum in organoid cultures may influence experimental outcomes, particularly in drug screening. While efforts have been made to co-culture tumor organoids with immune cells or fibroblasts to better mimic the tumor microenvironment, the cellular composition of these models remains markedly less complex than that of actual tissues. Incorporating functional vasculature and neural elements into organoid systems also remains a major obstacle. Moreover, studies indicated that tumor-derived organoids may exhibit abnormal cell division, resulting in slower proliferation rates compared to organoids derived from healthy tissues. Additionally, the current culture methodologies are not well-suited for modeling non-solid tumors. Overall, organoid research is still in its initial stages. Even for well-studied tissues such as the heart and liver, existing organoid models are relatively immature and provide only a partial representation of organ physiology. This issue is even more pronounced in complex systems like brain organoids. Advances in this field will require extensive research in areas such as cell biology and biochemistry and are likely to take considerable time [75,102].

The development and application of three-dimensional culture models in MM research have significantly advanced our understanding of the disease’s biology, tumor–stroma interactions, and therapeutic responses. However, despite their considerable promise, these models are still evolving. Future advancements will focus on refining the complexity of 3D systems to better replicate the full spectrum of the bone marrow microenvironment, including the interactions with immune cells, endothelial cells, and the dynamic extracellular matrix. Incorporating patient-derived samples and genetic heterogeneity will be crucial in creating more personalized models reflecting individual disease characteristics and responses to treatment. Furthermore, the integration of advanced technologies, such as organ-on-a-chip platforms, microfluidics, and real-time imaging, will enable more precise monitoring of cellular behavior and drug responses in 3D models, enhancing the predictive power of preclinical studies. As these models continue to evolve, they will undoubtedly play a pivotal role in advancing personalized medicine for MM, enabling the development of novel, targeted therapeutic strategies and improving the efficacy of existing treatments. Furthermore, the integration of 3D culture systems into the drug discovery process will facilitate the identification of new biomarkers and therapeutic targets, paving the way for more effective and tailored interventions for patients with MM.

## 6. CAR-T: A New Perspective

Among new perspectives, Chimeric Antigen Receptor T-Cell Immunotherapy (CAR-T therapy) is surely something to keep an eye on.

Tumor organoids, particularly patient-derived organoids (PDOs), closely resemble primary tumor tissues in terms of histopathological structure, genomic changes, and specific marker expression [103]. They have been widely used in areas such as drug screening, gene editing, and oncogene identification. Despite these advances, CAR-T therapy for solid tumors is still in an early exploratory phase. Tumor organoids provide distinct advantages over other preclinical models for CAR-T research, mainly because they preserve the biological features of primary tumors, which is essential for studying early-stage solid tumor CAR-T therapies. CAR-T are sure to improve existing therapies in the haematologyc field, especially for B-cell acute lymphoblastic leukemia (B-ALL), B-cell non-Hodgkin lymphoma (B-NHL), and multiple myeloma [104,105,106]. There is also an increased demand for personalized medicine, especially in regard to the efficacy of treatment.

Submerged Matrigel culture is a traditional and widely used technique for growing organoids, including tumor organoids. In this method, tumor tissue is first broken down into a single-cell suspension through enzymatic or mechanical means [99]. These isolated tumor cells are then embedded within a gel matrix and cultured in a medium that supports organoid development. The culture medium is formulated with essential nutrients, along with pathway inhibitors and/or growth factors, which are adjusted according to the specific type of tumor organoid being grown [107,108,109]. Common supplements include Wnt3a, R-spondin, epidermal growth factor (EGF), and Noggin—a bone morphogenetic protein (BMP) inhibitor—collectively promoting stem cell proliferation, differentiation, and self-renewal [110,111]. Enhancing CAR-T’s functionality to achieve long-lasting responses relies on a deep understanding of T cell biology, signaling pathways, and immunological memory. Gaining insight into the processes that drive the transition of resting naive T cells into rapidly dividing effector cells, and subsequently into resting memory cells, reveals critical factors that define an effective product [111]. This knowledge also highlights which cellular mechanisms can be modified to improve the overall phenotype. The field of CAR-T cell manufacturing is advancing rapidly, with automation becoming a key factor in streamlining production, increasing scalability, and addressing issues related to skill shortages and variability. Enhancing clinical outcomes depends on the thorough characterization of CAR-T products and involves not only technological innovations but also improved manufacturing approaches to prevent terminal differentiation. Accelerated manufacturing methods, such as next-day protocols, help overcome capacity constraints and are anticipated to significantly improve product effectiveness. Nonetheless, quality control remains a critical challenge that must be resolved to further shorten vein-to-vein times.

## 7. Conclusions

MM remains an incurable blood malignancy in which the dynamic crosstalk between malignant PCs and the bone marrow microenvironment plays a pivotal role in disease progression and therapy resistance. Although traditional 2D culture systems and in vivo models have yielded valuable insights, they fail in replicating the complex spatial and biochemical cues of the human bone marrow niche. Emerging 3D culture systems address these limitations by offering a more physiologically relevant context that recapitulates key aspects of the tumor microenvironment, including cell-to-cell and cell-to-matrix interactions, mechanical properties, and paracrine signaling. These systems demonstrated improved predictive power for drug efficacy and resistance mechanisms, such as modulating STAT3 activity and enhancing responses to agents like TRAIL and bortezomib. Advances in incorporating drug-loaded stromal cells and nanoparticle-based delivery within 3D constructs underscore their potential as platforms for precision drug testing and targeted therapies. Looking ahead, in future few years, standardized and clinically validated 3D models could become integral tools in translational research pipelines and preclinical screening, narrowing the gap between bench and bedside. Interestingly, patient-derived 3D cultures may enable real-time therapeutic profiling, helping clinicians to tailor regimens based on individual drug sensitivities and resistance landscapes. While challenges still remain—particularly in scalability, cost, and regulatory alignment—their successful integration could fundamentally reshape the therapeutic landscape of MM by accelerating drug development, reducing late-stage clinical trial failures, and delivering more personalized effective treatments.

## Figures and Tables

**Table 1:** A comparative analysis between 3D and 2D cell culture models.

Feature	2D Cell Culture Models	3D Cell Culture Models
Architecture	Monolayer, flat surface	Multicellular aggregates or organoids in a 3D scaffold
Cell–cell/cell–matrix interaction	Limited and artificial	More physiologically relevant
Mimicking bone marrow microenvironment	Poor	Good simulation of bone marrow niche
Drug response predictivity	Low predictive value for clinical outcomes	Improved correlation with in vivo responses
Immune interaction modeling	Absent or highly reduced	Potential to integrate immune components
Key pathway activation (e.g., STAT3)	Often not activated	STAT3 and other signaling pathways are activated as in vivo
Complexity and cost	Low cost, easy to handle	Higher cost, but more informative
Suitability for high-throughput screening	High	Moderate to high (increasing with organ-on-chip and automation)

**Table 2:** Summary of the advantages and limitations of static and dynamic cultures.

Aspect	Details
Limitations of 2D Culture	- Inadequate mimicry of in vivo conditions - Loss of cell-specific functions - Ineffective for complex drug response studies
Advantages of 3D Culture	- Mimics tissue architecture - Promotes natural cell interactions - Enhances differentiation, morphology, and gene expression
Static 3D Systems	Scaffold-free: - Hanging drop - Low-adhesion surfaces Scaffold-based: - Natural ECMs (e.g., collagen) - Hydrogels - Synthetic polymers - Ceramics
Dynamic 3D Systems	- Introduce mechanical forces (e.g., shear, tension) - Improve nutrient flow and cellular behavior - Better mimic physiological environments
Bioreactors and Microcarriers	- Enable large-scale cell culture - Used in vaccine and stem cell production - New designs improve cell harvest and viability
Organ-on-a-Chip	- Microfluidic platforms simulating organ function - Co-culture of multiple cell types - Useful for disease modeling and drug testing
Challenges	- Technical complexity and standardization issues - Difficulties in cell harvesting and scalability - Limited high-throughput compatibility
Future Directions	- Integrating 3D structure, dynamic cues, and co-culture - Customizable, scalable platforms for research and industry

**Table 3:** Three-dimensional models for biological research and medical applications come in various forms, each designed to mimic or support different aspects of natural tissue. Hydrogels provide a supportive environment for cells, making them useful for immune-related studies and healing. Spheroids and organoids are small, tissue-like clusters that simulate real tissues and immune responses. Three-dimensional bioprinted and nanostructured scaffolds allow precise control over shape and surface properties, helping to recreate complex tissue structures such as bone and blood vessels.

3D Model Structure	Materials/Scaffold	Biological Properties (Cells and Bioactivity)	Simulatable Dimensions
In situ vascularized bone via 3D bioprinting	GelMA hydrogel + BMSCs + endothelial cells in dual-extrusion printing with tubular channels	Promotes angiogenesis via endothelial sprouting; upregulates osteogenic genes; forms vascularized bone in vivo	Vascularization + bone regeneration
Dual-printed SLA + FDM vascularized bone	SLA-printed scaffold with PVA sacrificial channels + FDM PVA template	Mature co-culture of hMSCs and HUVECs; perfusable vessel formation; osteogenesis and angiogenesis coupling	Vascularization + bone differentiation
Cell-enhanced 3D-printed PCL/HAp scaffolds	3D-printed PCL/HAp scaffolds seeded with MSCs and EPC or HUVECs	In vitro vascular network; capillary infiltration and anastomosis in vivo; enhanced bone repair	Vascularization + bone regeneration
Nanofiber scaffold with osteo/angiogenic and immunomod	Xonotlite nanofiber + silk fibroin/gelatin hydrogel	Enhances BMSC osteo- and angiogenesis; reprograms macrophages to anti-inflammatory M2—promotes osteoimmune microenvironment	Vascularization + bone regeneration + immunomodulation
Nanoparticle-containing porous scaffold	Agarose + nanocrystalline apatite + VEGF-loaded nanoparticles	Nanoparticles induce M2 macrophage polarization, promote IL-10 secretion, and support MSC osteogenesis and angiogenesis in vitro and in vivo	Vascularization + bone support + immunomodulation
3D-printed GelMA-based hydrogel (multifunctional)	GelMA-CQD or GelMA-PPy-Fe in extrusion-based 3D printing	M2 macrophage polarization; anti-inflammatory; enhanced osteogenesis and angiogenesis; shows tumor-combatting + bone repair properties	Vascularization + bone regeneration + immunomodulation

**Table 4:** Spheroids provide physiological gradients and are ideal for drug screening, though they lack matrix interactions and show size variability. Organoids better recapitulate tissue complexity via ECM scaffold support but rely heavily on Matrigel, which has batch variability. Hydrogel scaffolds (especially hybrid hydrogels) allow control over mechanical and biochemical cues; synthetic variants improve reproducibility but require functionalization. Organ-on-chip platforms simulate flow, perfusion, and multicellular interactions; recent advances include lung- and liver-chip systems. Three-dimensional bioprinting enables precisely patterned constructs like liver discoids that display in vivo-like albumin/urea production and ADME profiling.

3D Model	Materials/Scaffold	Cell Sources	Bio-Functional Evaluation Metrics	Advantages/Limitations
Spheroids (scaffold-free)	No scaffold; use ultra-low attachment plates, hanging drop, spinner, magnetics	Cell lines, primary cells, co-cultures	Oxygen/nutrient gradient formation; necrotic core; drug response assays	Simple, scalable, easy, and cost-effective; however, there is size variability and mechanical/mechano-biological limitations
Organoids	ECM-based hydrogels (e.g., Matrigel), natural or synthetic hydrogels	Stem cells (iPSC/ESC), primary tissue	Structural organization, differentiation, gene/protein profiling, multi-cell type presence	Recapitulates organ complexity, heterogeneity; there is batch variability (Matrigel) and costly, time-intensive protocols
Hydrogel-based scaffolds	Natural (collagen, HA, alginate) or synthetic (PEG, nanocellulose)	Cell lines, primary cells, iPSC	Viability/growth, mechanical properties (rheology), adhesion, differentiation	ECM-like environment, tunable mechanics; natural gels are variable, synthetics may lack bioactivity
Organ-on-chip/microfluidics	PDMS, glass; integrated ECM like collagen, decellularized ECM	Primary cells, tumor, endothelial, immune	Fluid flow, barrier function, migration, toxicity response, multi-tissue interaction	Mimics physiological flow and dynamics, vascularization; complex, costly, low throughput
3D bioprinting	Bio-inks: cell-laden hydrogels (PEG, collagen, gelatin), microgels	Cell lines, primary cells, stem cells	Structural precision, viability, function (e.g., albumin production), ADME gene benchmarking	Highly customizable and scalable; expensive equipment, bio-ink formulation challenges

## Abbreviations

The following abbreviations are used in this manuscript:

WHO	World Health Organization.
MM	Multiple myeloma.
PCs	Plasma cells.
FDM	Fused deposition modeling.
SLA	Stereolithography.
DIW	Direct ink writing.
LGDW	Laser-guided direct writing.
PCL	Polycaprolactone.
PLA	Poly(lactic acid).
MGUS	Monoclonal gammopathy of undetermined significance.
TNF-α	Tumor necrosis factor alpha.
IL-1	Interleukin 1.
TEM	Effector memory T.
TEMRA	Terminally differentiated effector memory RA.
MDSCs	Myeloid-derived suppressor cells.
PFS	Progression free survival.
OS	Overall survival.
T-regs	Regulatory T cells.
TGF-β	Transforming growth factor beta.
SDF-1	Stromal cells derived factor 1.
IMiDs	Immunomodulatory drugs.
CAR-T	Chimeric antigen receptor T-cell.
TME	Tumor microenvironment.
ASCs	Adult stem cells.
ESCs	Embryonic stem cells.
iPSCs	Induced pluripotent stem cells.
CLL	Chronic lymphocytic leukemia.
BM	Bone marrow.
RCCS™	Rotary cell culture system.
HS-5	Human bone marrow stromal cell.
HARV	High aspect ratio vessels.
ECM	Extracellular matrix.
WNT	Wingless/integrated.
APC	Antigen-presenting cell.
GSK-3β	Glycogen synthase kinase-3 beta.
CK1	Casein kinase 1.
FZD	Frizzled.
LRP5/6	Lung resistance-related protein.
PCP	Planar cell polarity.
ROR2	Receptor tyrosine kinase-like orphan receptor 2.
PI3K	Phosphoinositide 3-kinase.
AKT	Protein kinase B.
mTOR	Mammalian target of rapamycin.
EMD	Extramedullary.
STAT3	Signal transducer and activator of transcription 3.
ERK 3	Extracellular signal-regulated kinase.
MAPK	Mitogen-activated protein kinase.
NFKb	Nuclear Factor kappa-light-chain-enhancer of activated B cells
EGFR	Epidermal growth factor receptor.
SAHA	Suberoylanilide hydroxamic acid.
HDAC	Histone deacetylase inhibitor.
TRAIL	Tumor necrosis factor-related apoptosis-inducing ligand.
MSCs	Mesenchymal stromal cells.
BM-MSCs	Bone marrow mesenchymal stromal cells.
PTX	Paclitaxel.
CAM-DR	Cell adhesion-mediated drug resistance.
VEGF	Vascular endothelial growth factor.
RAF	Rapidly accelerated fibrosarcoma.
MEK	Mitogen-activated protein kinase.
CRISPR-Cas9	Clustered regularly interspaced short palindromic repeats and CRISPR-associated protein 9.
DMEM	Dulbecco’s Modified Eagle Medium.
EGF	Epidermal growth factor.
FGF10	Fibroblast growth factor 10.
HEPES	2-[4-(2-hydroxyethyl)piperazin-1-yl]ethanesulfonic acid.
HGF	Hepatocyte growth factor.
